# Self-management interventions in patients with long-term conditions: a structured review of approaches to reporting inclusion, assessment, and outcomes in multimorbidity

**DOI:** 10.15256/joc.2014.4.33

**Published:** 2014-08-28

**Authors:** Cassandra Kenning, Peter A. Coventry, Peter Bower

**Affiliations:** ^1^NIHR School for Primary Care Research, Centre for Primary Care, Manchester Academic Health Science Centre, University of Manchester, Manchester, UK; ^2^NIHR Collaboration for Applied Health Research and Care – Greater Manchester, Manchester Academic Health Science Centre, University of Manchester, Manchester, UK; ^3^NIHR Greater Manchester Primary Care Patient Safety Translational Research Centre, Centre for Primary Care, Manchester Academic Health Science Centre, University of Manchester, Manchester, UK

**Keywords:** Comorbidities, multimorbidity, multiple chronic conditions, self-management interventions, review, external validity

## Abstract

**Background:**

Multimorbidity has many potential implications for healthcare delivery, but a particularly important impact concerns the validity of trial evidence underpinning clinical guidelines for individual conditions.

**Objective:**

To review how authors of published trials of self-management interventions reported inclusion criteria, sample descriptions, and consideration of the impact of multimorbidity on trial outcomes.

**Methods:**

We restricted our analysis to a small number of exemplar long-term conditions: type 2 diabetes mellitus, coronary heart disease, and chronic obstructive pulmonary disease. We focussed our search on published Cochrane reviews. Data were extracted from the trials on *inclusion/exclusion*, *sample description*, and *impact on outcomes*.

**Results:**

Eleven reviews consisting of 164 unique trials were identified. Sixty percent of trials reported excluding patients with forms of multimorbidity. Reasons for exclusion were poorly described or defined. Reporting of multimorbidity within the trials was poor, with only 35% of trials reporting on multimorbidity in their patient samples. Secondary analyses, exploring the impact of multimorbidity, were very rare.

**Conclusions:**

The importance of multimorbidity in trials is only going to become more important over time, but trials often exclude patients with multimorbidity, and reporting of multimorbidity in trials including such patients is generally poor. This limits judgements about the external validity of the results for clinical populations. A consistent approach to the conduct and reporting of secondary analyses of the effects of multimorbidity on outcomes, using current best-practice guidance, could lead to a rapid development of the evidence base.

## Introduction

Care for patients with long-term conditions is generally designed around the individual long-term conditions, whereas many patients in primary care have multimorbidity [1, 2]. Key issues in the literature surrounding multimorbidity and its inclusion/reporting in trials are that of definition and measurement. Whilst there has been some debate around the terms used: multimorbidity defined as “the co-existence of two or more chronic conditions, where one is not necessarily more central than the others” [3] versus comorbidity which implies an index condition to which coexistent conditions relate or share an aetiology [4]. There are also potential issues around how multimorbidity or comorbidity are measured – either by using restricted lists of conditions (and different studies may use different lists) or whether all possible conditions are included. Added to this, there may also be differences as to which conditions are considered to qualify as long-term conditions (e.g. hypertension, hypercholesterolaemia, and high blood pressure may or may not be included as “long-term” conditions). Therefore, defining and clearly describing patient samples in terms of the number of long-term conditions they have is extremely complex and lacks clear, agreed, and standardized reporting procedures.

Multimorbidity has many effects on patient experience and outcomes, but a particularly important impact of multimorbidity relates to the validity of trial evidence underpinning clinical guidelines for individual conditions. There is some evidence that patients with multimorbidity are routinely excluded from many trials [5, 6]. The impact of any particular intervention may be increased or decreased in patients with multimorbidity, and trial results (and the resulting guidelines) may lack external validity [7–9].

There are three main ways in which the presence of multimorbidity may impact on the external validity of trials [10].

1. *Inclusion/exclusion*: patients with multimorbidity may be excluded from trials. This will impact on the external validity of the results and the degree to which they can be applied to clinical populations exhibiting high levels of multimorbidity.
2. *Sample description*: trials may present data on rates and types of multimorbidity, which allows some assessment of the degree to which the results can be generalized to other populations.
3. *Impact on outcomes*: trials may present secondary or moderator analyses exploring the impact of the intervention on patients with and without multimorbidity.

### Self-management as an exemplar

Self-management is increasingly seen as being key to the effective management of long-term conditions [11, 12], because of the importance of health behavior in long-term conditions, and because of the potential for self-management to provide savings in healthcare costs. Multimorbidity may have particular implications for trials of self-management interventions, as these patients face complex management regimes [13] and difficult decisions about priorities [14]. Patients with multimorbidity are also likely to demonstrate characteristics which will further limit self-management, such as poor general health [3], advanced age [15], cognitive impairment [16], and low health literacy [17]. All these factors make it possible that effective self-management interventions may be less effective in patients with multimorbidity. The opposite effect is also possible. Patients with multimorbidity may have the greatest capacity to benefit, because their baseline health is poor, and because there are potential synergies in terms of management (e.g. several conditions may benefit from increased exercise or a better diet). However, another factor to consider is that of treatment burden. Patients with multiple conditions often have complex treatment regimens with little co-ordination between treatment and services for different conditions. Shifting the management of chronic diseases from the clinic to the home may present a considerable burden for some patients with multimorbidity [18]. Currently there is no clear evidence that self-management interventions are effective in patients with multimorbidity, which, in part, may be due to the way in which self-management trials are being reported.

The potential impacts discussed above may have important implications. If self-management interventions are *less* effective in those patients with multimorbidity, it is critical that new interventions are developed and evaluated to meet their needs. If existing interventions demonstrate equal or greater effectiveness in patients with multimorbidity, then there is a need to ensure that services prioritize referral and support for patients with multimorbidity to ensure that they achieve these benefits.

A number of published articles have explored how trials currently report and treat multimorbidity in disease-specific intervention trials [8, 9]. The current study explores how authors of published trials of self-management interventions have managed inclusion, sample description, and consideration of the impact of multimorbidity on outcomes within trials. This article assesses current approaches to these issues and considers implications for evidence synthesis, and future trial reporting and analysis.

## Materials and Methods

To achieve our aims, we restricted our analysis to three exemplar conditions, where multimorbidity is common [19] and where there is a known self-management literature. The disorders chosen were type 2 diabetes mellitus (DM), coronary heart disease (CHD), and chronic obstructive pulmonary disease (COPD). Furthermore, we restricted our initial search to published Cochrane reviews. Many of the core self-management interventions in our exemplar groups have already been assessed through the Cochrane review process. Restricting our search to published Cochrane reviews reduced the scope of the review to a manageable size, but ensured that the source reviews were themselves of consistent quality.

### Search strategy

We searched the Cochrane Database of Systematic Reviews using a standardized list of search terms for self-management interventions developed for a previous study (see Supplementary Table 1). We defined a self-management support intervention as “one primarily designed to develop the abilities of patients to undertake management of health conditions through education, training, and support to develop patient knowledge, skills, or psychological and social resources”.

One researcher made assessments about the eligibility of the reviews in terms of disease and self-management interventions. We identified all unique trials within those reviews, and accessed the full text of those trials to conduct data extraction. Non-English-language papers were excluded because of a lack of funds for translation. A second researcher cross-checked the reviews for inclusion, and a 5% sample of the 164 unique trials.

### Data extraction and analysis

We extracted data from each trial within the included reviews, on patient inclusion, assessment, and outcome, as it is related to conditions other than the index condition. We present a narrative description of reporting in relation to multimorbidity in the included trials, in terms of:

1. *Inclusion/exclusion*: proportion of studies excluding patients with multimorbidity at baseline. Any condition-specific exclusions and reasons for exclusions were extracted.
2. *Sample description*: reporting of patient characteristics in terms of multimorbidity.
3. *Impact on outcome*: (i) analyses of the impact of multimorbidity on outcomes through secondary and moderator analyses, or (ii) analysis of outcomes on comorbid conditions.

## Results

**Figure 1:**
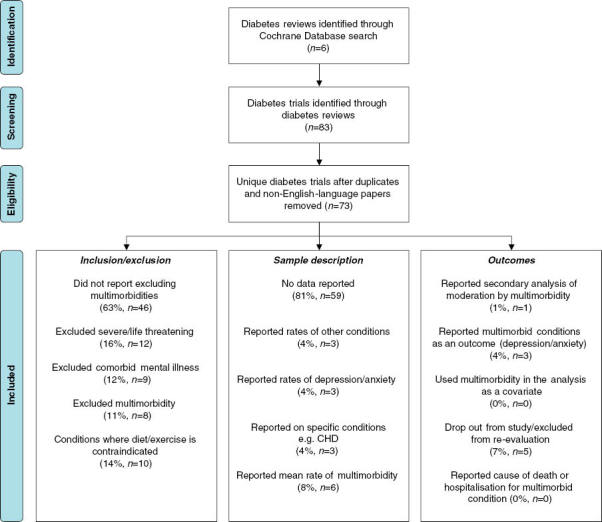
Modified PRISMA (Preferred Reporting Items for Systematic Reviews and Meta-Analyses) diagram for type 2 diabetes mellitus (DM). The diagram shows the number of Cochrane reviews identified (*n*=6), the total number of trials within those reviews (*n*=83), and the number of unique trials once duplicate and non-English-language papers were removed (*n*=73). The diagram also records the data extracted from the studies in terms of our three aims.

Modified PRISMA (Preferred Reporting Items for Systematic Reviews and Meta-Analyses) diagrams have been used to illustrate the number of trials identified from Cochrane reviews and the number of included trials reporting on multimorbidity. See Figure 1 (DM trials), 2 (CHD trials), and 3 (COPD trials). The figures show the number of Cochrane reviews identified, the total number of trials within those reviews, and the number of unique trials once duplicates (*n*=10; 5%) and non-English-language papers (*n*=9; 5%) were removed. The figures also record the data extracted from the studies in terms of our three domains. Further information on the number of Cochrane reviews screened for each condition and the reasons for excluding reviews is available in the Supplementary Materials and Methods. A list of the included Cochrane reviews is provided in Supplementary Table 2.

### Inclusion/exclusion

In total, across conditions, 60% of trials reported excluding patients with multimorbidity. However, the number of studies excluding patients based on multimorbidity was not equal across conditions. The majority of DM trials (*n*=46; 63%) included patients with multimorbidity. In contrast, the number of CHD and COPD studies that included patients with multimorbidity was much lower, 20% (*n*=11) and 22% (*n*=8), respectively. The main exclusions were of patients where diet/exercise was contraindicated for their health or where they were unable to take part in general physical activity (COPD, 44%; CHD, 40%; DM, 14%). Patients with severe or life-threatening conditions were also frequently excluded (CHD, 31%; COPD, 22%; DM, 16%) although inclusion in this category and how it was assessed was generally not defined by the authors. Some studies also excluded comorbid mental illness (CHD, 14.5%; DM, 12%; COPD, 8%), the parameters of which were generally left undefined.

We tried to quantify the impact of excluding patients with multimorbidity on recruitment rates for the trials. However, only 21% of trial papers reported the actual number of potential participants who were excluded due to multimorbidity (as separate from an overall number of patients excluded for any reason). As with the reporting of exclusion criteria and sample descriptions, the detail available from CHD trials (*n*=14) was much better than that of COPD (*n*=6), and DM (*n*=1). As a percentage of the total participants screened for these trials, where data were available, 22.5% (3,915/17,417) of patients were excluded due to multimorbid conditions. Percentages of patients excluded from trials due to multimorbid conditions ranged from 4% to 60% of total screened participants.

### Sample description

Reporting of multimorbid conditions within the trials was lacking in most cases, with only 35% of trials reporting on multimorbidity in their patient samples. Again, levels of reporting differed across conditions. Only 19% of DM trials reported any other long-term conditions within their patient samples. CHD trials were much more likely to report multimorbidity than DM trials, with 58% reporting on multimorbid conditions within their patient samples. COPD trials also had better reporting rates than DM trials, with 33% of trials reporting on some multimorbid conditions. As stated previously, a significant proportion of trials reported including patients with multimorbidity. One might expect that trials including patients with multimorbidity would be more likely to report rates of multimorbidity in their sample data. However, of the 65 trials that included patients with multimorbidity, only 25% (*n*=16) reported on multimorbidity in their patient samples. The majority of those trials that did report multimorbidity reported a mean number of multimorbid conditions (*n*=6): very few reported the rates of specific conditions.

### Impact on outcome

In total, across conditions, just three trials (1.8%) reported secondary analysis of multimorbidity as a moderator [20–22]. Three trials reported on impact of the intervention on comorbid depression/anxiety [23–25], and two trials used multimorbidity in the analysis as a covariate [26, 27].

Only one DM trial conducted moderator analyses to evaluate potential interaction effects on all outcomes, which included number of multimorbid conditions [20]. The authors reported that all moderator analyses were non-significant at *p* <0.01. There were three DM trials [23–25] that included anxiety/depression as an outcome variable and therefore reported on intervention impact on comorbid anxiety/depression. Only three CHD trials reported the inclusion of multimorbidity in their analyses [21, 26, 27]. Peikes et al. controlled for prior diagnoses on 10 other chronic conditions, in their regression modeling [21]. Clark et al. measured impact on a range of symptoms and concluded that patients experienced less impact of all types of symptoms, that is, symptoms beyond just those associated with their heart condition [26]. In their analysis, Zwisler et al. reported that after adjusting for age and comorbidity, mortality was almost twice as high among the non-participants compared with the participants at 12 months (relative risk, 1.87; 95% confidence interval [CI], 1.19–2.85) [27]. Only one COPD trial reported any secondary analysis for moderation by multimorbidity. Blake and colleagues stated that baseline multimorbidity was included as an independent variable in the analysis of morbidity outcomes [22]. However, this trial does not report the outcome of these analyses, but concludes that it is unlikely that comorbidity confounded the results.

## Discussion

### Summary

The aim of this research was to determine how authors of published trials of self-management interventions have managed inclusion, description, and impact of multimorbidity in exemplar disorders. Although it is often assumed that many clinical trials exclude patients with multimorbidity, we found that many trials did not exclude patients with multimorbidity, although many trials failed to clearly report their definitions of conditions that were excluded. Mental health problems, such as depression, are highly prevalent in patients with multimorbidity, and there is evidence that the combination of depression and other long-term conditions is particularly problematic [28]. It is noteworthy that the lack of detail about multimorbidity as an exclusion was particularly evident in terms of mental illness: there were no descriptions about what was classed as a mental illness, severity of the illness, or whether the illness was current or patients had a history of illness, and also about how this information was obtained.

In terms of sample descriptions, the information that the authors collected/reported was very limited. Many trials consistently failed to report levels of multimorbidity within their included patient samples, which limits the ability of the reader to assess external validity of trial results. Particularly noteworthy were those trials that stated they had not excluded patients based on multimorbidity, but then gave no information at all about other long-term conditions in their patient samples. Finally, the impact of multimorbidity as a moderator of treatment effect was very rare and inconsistently reported. This means that it was not possible to draw any conclusions about the impact of multimorbidity on the effectiveness of self-management interventions.

### Literature

Our results support previous work that identifies the problem of lack of clear reporting of patient characteristics in clinical trials [29]. They also support the view that there is a lack of published information, needed to support clinical decision-making, in patients with multimorbidity [7, 30]. The review carried out by Boyd et al. focussed on both drug therapies and those which implemented a diet or exercise interventions in patients with COPD, heart failure, type 2 DM, or stroke [30]. The authors of the review reported that the replicability of both inclusion and exclusion was only moderate. They also showed that the reporting of multimorbidity was very limited, with only 43.5% of trials describing the prevalence of any comorbidity [30]. This figure is similar to our results in which only 35% of trials described the prevalence of any comorbidity.

Research by Fortin et al. looked at how patient characteristics were reported in five randomly selected hypertension trials. They stated that none of the five trials reported how many patients with comorbidity were excluded or how many patients participated after meeting the inclusion criteria [7]. Applying the same inclusion criteria to their own patient database, the percentage of eligible patients who also had comorbidity ranged from 89% to 100%, and the mean number (±standard deviation) of chronic conditions among patients ranged from 5.5±3.3 to 11.7±5.3 [7]. Their results show that given a general primary-care population most, if not all, patients in a trial sample have comorbidity, but trials are not reporting these data.

Unlike the Boyd et al.’s review [30], our review focusses specifically on self-management interventions. We chose self-management programmes because self-management is the recommended clinical practice for patients with long-term conditions, and because there are good theoretical and empirical reasons why multimorbidity would be particularly important in these conditions. To be clinically useful, the results of trials must be relevant to definable groups of patients in particular settings [5]. However, as we describe, it is not always easy to find the information needed to do this in trial reports. As it has previously been noted, publication of trial methods, the analyses carried out, and the information reported is at the discretion of the authors [31]. As was found in the review by Ross et al., key analyses that might have been conducted to better inform clinical practice, such as secondary analysis of moderator effects, are often not conducted, or not reported by trial authors [31]. Improved data-sharing from trials may improve clarification of trial participants and allow further analyses into the impact of multimorbidity on patient outcomes [31].

### Strengths/limitations

Trials and reviews are used to inform clinical decision-making, and our study explored how these trials managed the issue of multimorbidity. It looked at how this is dealt with, within and across major long-term conditions where multimorbidity is common [32]. The size and scope of the existing self-management literature is such that a comprehensive search across all conditions and interventions was beyond the scope and resources of the current review. However, we feel that restriction to Cochrane reviews made sense, to ensure a consistent level of quality. The three exemplar conditions were chosen because they are conditions that are likely to be helped by, and are therefore linked with, self-management interventions. Rates of comorbidity and multimorbidity are also high in these conditions. However, limiting the review to just three exemplar conditions may mean that there is a body of literature for self-management interventions in other long-term conditions that does adequately consider the impact and implications of multimorbidity on the external validity of the results, but we feel that this is unlikely. Another limitation to the scope of the study was the exclusion of trials not published in the English language (*n*=9). We did not have the resources for the translation of these articles, but this represented a small number of trials (5% of the unique trials) so we would not expect this to have impacted the results or conclusions drawn by this review.

### Informing research/clinical practice

Self-management interventions are increasingly being promoted for the care and maintenance of patients with long-term conditions. Strategies tend to focus on providing knowledge to patients about their condition(s) and to promoting healthy behaviors, such as diet and exercise. Due to either the exclusion of patients with multimorbidity, the poor reporting of patient sample characteristics, and the lack of analysis of moderators, it is not possible for clinicians to make informed decisions about whether a self-management intervention is an appropriate intervention for a particular patient.

Despite the high prevalence of multimorbidity, treatment guidelines have continued to be based on trials that either exclude patients with multimorbidity or fail to report clear descriptions of the patient sample, because this is all that is available. A recent press release by the chairman of NICE (National Institute for Health and Care Excellence) states that new more clinically relevant guidelines taking into account the complexity of patients seen in general practice are being developed [33]. However, as this review has shown, it will be difficult to make recommendations about the appropriateness of interventions for patients with multimorbidity, based on the current published research.

#### Inclusion/exclusion

Sample selection is often restricted to those most likely to respond to an intervention and by the need to achieve high internal validity. This type of study may be good to test initial efficacy but is insufficient to determine treatment recommendations and should be followed by effectiveness studies that use patient samples more representative of clinical populations. However, further effectiveness trials are rarely being conducted in self-management intervention studies.

#### Sample descriptions

As described in our results, trials do not always exclude patients with multimorbidities, and so if the patient samples were more clearly described in these trials, any positive results should be replicable in normal clinical populations. Poor reporting of patient characteristics in terms of multimorbidity means that it is not easy to draw conclusions about the effectiveness of self-management interventions in patients with multiple long-term conditions, and there is no clear evidence of whether multimorbidity impacts on the outcomes of self-management interventions [6].

Better reporting of sample demographics and baseline data as a standard, would make it easier to identify whether patient samples used in trials are representative of normal clinical populations, although it is dependent on agreed systems for defining and reporting multimorbidity [1, 32, 34].

#### Impact on outcomes

The lack of secondary analyses on trial results means it is not possible to determine whether self-management interventions are particularly good for those with multimorbidity, i.e., they have benefitted the most or conversely, they have not benefitted from the intervention as much as those with a single condition. A consistent approach to the conduct and reporting of secondary analyses of the effects of multimorbidity on outcomes, using current best-practice guidance [35, 36], could lead to a rapid development of the evidence base.

Improving data-sharing between clinical trials could significantly improve the information we have on the impact of multimorbidity on self-management interventions. The availability of archived datasets [31] does provide a platform for researchers to conduct individual patient data analyses on moderator variables, such as multimorbidity [10, 37]. However, there may be significant limits to the ability to “recover” data about multimorbidity from existing datasets, and the main impact on better conduct and reporting may be in trials going forward. Datasets like the planned “care.data” [38] will allow researchers to observe the effects of multimorbidity on newly introduced interventions. Rapid action on this issue is thus required if improvements are to be made.

## Conclusions

A proportion of self-management intervention trials exclude patients with multimorbidity, even though patients with multimorbidity are the most common in clinical practice. Trials need to be more inclusive to improve external validity of trial results. The current standard for reporting multimorbidity in self-management intervention participant samples is poor. Improved reporting of sample demographic data and secondary analyses for any potential moderator effect of multimorbidity is needed in order to assess the utility of the evidence base for self-management interventions on patients with multimorbidity.

## Figures and Tables

**Figure 2:**
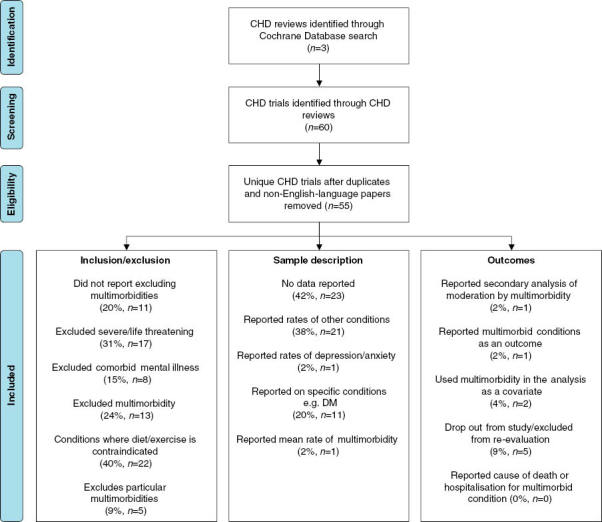
Modified PRISMA (Preferred Reporting Items for Systematic Reviews and Meta-Analyses) diagram for coronary heart disease (CHD). The diagram shows the number of Cochrane reviews identified (*n*=3), the total number of trials within those reviews (*n*=60), and the number of unique trials once duplicates and non-English-language papers were removed (*n*=55). The diagram also records the data extracted from the studies in terms of our three aims.

**Figure 3:**
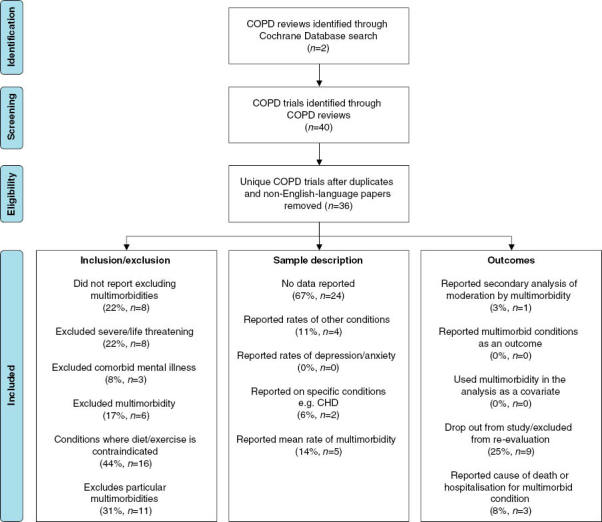
Modified PRISMA (Preferred Reporting Items for Systematic Reviews and Meta-Analyses) diagram for chronic obstructive pulmonary disease (COPD). The diagram shows the number of Cochrane reviews identified (*n*=2), the total number of trials within those reviews (*n*=40), and the number of unique trials once duplicate and non-English-language papers were removed (*n*=36). The diagram also records the data extracted from the studies in terms of our three aims.

## References

[1] Salisbury C, Johnson L, Purdy S, Valderas J, Montgomery A (2011). Epidemiology and impact of multimorbidity in primary care: a retrospective cohort study. Br J Gen Pract.

[2] Barnett B, Mercer S, Norbury M, Watt G, Wyke S, Guthrie B (2012). The epidemiology of multimorbidity in a large cross-sectional dataset: implications for health care, research and medical education. Lancet.

[3] Boyd C, Fortin M (2011). Future of multimorbidity research: how should understanding of multimorbidity inform health systems design?. Public Health Rev.

[4] Smith SM, O’Dowd T (2007). Chronic diseases: what happens when they come in multiples?. Br J Gen Pract.

[5] Rothwell P (2005). External validity of randomised controlled trials: to whom do the results of this trial apply?. Lancet.

[6] Richardson WS, Doster LM (2014). Comorbidity and multimorbidity need to be placed in the context of a framework of risk, responsiveness and vulnerability. J Clin Epidemiol.

[7] Fortin M, Dionne J, Pinho G, Gignac G, Almirall J, Lapointe L (2006). Randomized controlled trials: do they have external validity for patients with multiple comorbidities?. Ann Fam Med.

[8] Steg PG, Lopez-Sendon J, Lopez de Sa E, Goodman SG, Gore JM, Anderson FA (2007). External validity of clinical trials in acute myocardial infarction. Arch Intern Med.

[9] Travers J, Marsh S, Caldwell B, Williams M, Aldington S, Weatherall M (2007). External validity of randomized controlled trials in COPD. Respir Med.

[10] Fortin M, Smith S (2013). Improving the external validity of clinical trials: the case of multiple chronic conditions. J Comorbidity.

[11] Bodenheimer T, Wagner E, Grumbach K (2002). Improving primary care for patients with chronic illness: the Chronic Care Model, part 2. J Am Med Assoc.

[12] Bodenheimer T, Lorig K, Holman H, Grumbach K (2002). Patient self-management of chronic disease in primary care. J Am Med Assoc.

[13] Lin E, Katon W, Von Korff M, Tang L, Williams J, Kroenke K (2003). Effect of improving depression care on pain and functional outcomes among older adults with arthritis. J Am Med Assoc.

[14] Fried T, McGraw S, Agostini J, Tinetti M (2008). Views of older persons with multiple morbidities on competing outcomes and clinical decision making. J Am Geriatr Soc.

[15] Vogeli C, Shields A, Lee T, Gibson T, Marder W, Weiss K (2007). Multiple chronic conditions: prevalence, health consequences and implications for quality, care management and costs. J Gen Intern Med.

[16] van Dijk P, Mehr D, Ooms M, Madsen R, Petroski G, Frijters D (2005). Comorbidity and 1-year mortality risks in nursing home residents. J Am Geriatr Soc.

[17] Nutbeam D (2008). The evolving concept of health literacy. Soc Sci Med.

[18] May C, Montori V, Mair F (2009). We need minimally disruptive medicine. Br Med J.

[19] Weiss CO, Boyd CM, Yu Q, Wolff JL, Leff B (2007). Patterns of prevalent major chronic disease among older adults in the United States. J Am Med Assoc.

[20] Glasgow RE, Nutting PA, Toobert DJ, King DK, Strycker LA, Jex M (2006). Effects of a brief computer-assisted diabetes self-management intervention on dietary, biological and quality-of-life outcomes. Chronic Illn.

[21] Peikes D, Chen A, Schore J, Brown R (2009). Effects of care coordination on hospitalization, quality of care, and health care expenditures among medicare beneficiaries: 15 randomized trials. J Am Med Assoc.

[22] Blake RL, Vandiver TA, Braun S, Bertuso DD, Straub V (1990). A randomised controlled evaluation of a psychosocial intervention in adults with chronic lung disease. Fam Med.

[23] Rosal MC, Olendzki B, Reed GW, Gumieniak O, Scavron J, Ockene I (2005). Diabetes Self-Management Among Low-Income Spanish-Speaking Patients:A Pilot Study. Ann Behav Med.

[24] Siebolds M, Gaedeke O, Schwedes U (2006). Self-monitoring of blood glucose-psychological aspects relevant to changes in HbA1c in type 2 diabetic patients treated with diet or diet plus oral antidiabetic medication. Patient Educ Couns.

[25] Lim S, Kang SM, Shin H, Lee HJ, Yoon JW, Yu SH (2011). Improved glycemic control without hypoglycemia in elderly diabetic patients using the ubiquitous healthcare service: a new medical information system. Diabetes Care.

[26] Clark NM, Janz NK, Dodge JA, Xihong L, Trabert BL, Kaciroti N, et al (2009). Heart Disease Management by Women: does intervention format matter?. Health Educ Behav.

[27] Zwisler ADO, Soja AMB, Rasmussen S, Frederiksen M, Abadini S, Appel J (2008). Hospital-based comprehensive cardiac rehabilitation versus usual care among patients with congestive heart failure, ischemic heart disease, or high risk of ischemic heart disease: 12-month results of a randomized clinical trial. Am Heart J.

[28] Moussavi S, Chatterji S, Verdes E, Tandon A, Patel V, Ustun B (2007). Depression, chronic disease, and decrements in health: results from the World Health Surveys. Lancet.

[29] Smith SM, Bayliss EA, Mercer SW, Gunn J, Vertergaard M, Wyke S (2013). How to design and evaluate interventions to improve outcomes for patients with multimorbidity. J Comorbidity.

[30] Boyd, CM, Vollenweider D, Puhan MA (2012). Informing evidence-based decision-making for patients with comorbidity: availability of necessary information in clinical trials for chronic diseases. PLoS One.

[31] Ross JS, Lehman R, Gross CP (2012). The importance of clinical trial data sharing toward more open science. Circ Cardiovasc Qual Outcomes.

[32] Huntley A, Johnson R, Purdy S, Valderas J, Salisbury C (2012). Measures of multimorbidity and morbidity burden for use in primary care and community settings: a systematic review and guide. Ann Fam Med.

[33] Price C NICE to move to multimorbidity guidance, chief says.

[34] Valderas J, Starfield B, Salisbury C, Sibbald B, Roland M (2009). Defining comorbidity: implications for the understanding and provision of health services and health. Ann Fam Med.

[35] Pincus T, Miles C, Froud R, Underwood M, Carnes D, Taylor S (2011). Methodological criteria for the assessment of moderators in systematic reviews of randomised controlled trials: a consensus study. BMC Med Res Methodol.

[36] Sun X, Briel M, Busse J, You J, Akl E, Mejza F (2012). Credibility of claims of subgroup effects in randomised controlled trials: systematic review. Br Med J.

[37] Bower P, Kontopantelis E, Sutton A, Kendrick T, Richards D, Gilbody S (2013). Influence of initial severity of depression on effectiveness of low intensity interventions: meta-analysis of individual patient data. Br Med J.

[38] NHS England The care.data programme – better information means better care.

